# Supplementary material for: ROCK and the actomyosin network control biomineral growth and morphology during sea urchin skeletogenesis
Source: eLife. 2024 Apr 4;12:RP89080. doi: 10.7554/eLife.89080 (PMC10994658; doi:10.7554/eLife.89080)
Supplement: Supplementary file 1. — (a) Rho-associated coiled-coil kinase (ROCK) inhibition experimental details. The table provides the number of biological replicates and embryos scored for each experiment with ROCK inhibitor. (b) µCT statistics of control and ROCK inhibited spicules. The table provides the details of the number of spicules measured, average and standard deviation of measured length, thickness, volume, and surface area. (c) µCT statistical significance. The table provides the statistical significance between µCT measurements of control and ROCK-inhibited spicules at 2dpf and 3dpf, based on the parametric student’s T-test. (d) Lantrunculin-A and Blebbistatin experimental details. The table provides the number of biological replicates and embryos scored for each experiment with Lantrunculin-A and Blebbistatin. [file elife-89080-supp1.docx]

**Supplementary files for -** **ROCK and the actomyosin network control biomineral growth and morphology during sea urchin skeletogenesis**

Eman Hijaze^1^, Tsvia Gildor^1^, Ronald Seidel^2,&^, Majed Layous^1^, Mark Winter^3^, Luca Bertinetti^2^, Yael Politi^2^ and Smadar Ben-Tabou de-Leon^1,^*

^1^Department of Marine Biology, Leon H. Charney School of Marine Sciences, University of Haifa, Haifa 31905, Israel.

^2^B CUBE Center for Molecular Bioengineering, Technische Universität Dresden, 01309 Dresden, Germany.

^3^Department of Electrical Engineering, Computer Science and Mathematics, Technische Universiteit Delft, Delft 2628CD, Netherlands

^&^Current address: Section Biomedical Imaging, Molecular Imaging North Competence Center (MOIN CC), Dept. Radiology and Neuroradiology, University Medical Center Kiel, Kiel University, 24118 Kiel, Germany.

*Correspondence: Smadar Ben-Tabou de-Leon ([sben-tab@univ.haifa.ac.il](mailto:sben-tab@univ.haifa.ac.il))

**This PDF file includes:** Supplementary files 1a-1d

**Supplementary file 1a**

| **Condition** | **# replicates** | **# embryos scored** |
| --- | --- | --- |
| DMSO (control) | 8 | 438 |
| Y0 10µM | 3 | 122 |
| Y25 10µM | 3 | 109 |
| Y0 30µM | 3 | 120 |
| Y20 30µM | 3 | 103 |
| Y25 30µM | 3 | 108 |
| y0 40µM | 3 | 93 |
| y20 40µM | 3 | 123 |
| y25 40µM | 3 | 101 |
| y0 80µM | 8 | 377 |
| y20 80µM | 3 | 116 |
| y25 80µM | 7 | 369 |
| Wash 80µM | 5 | 268 |

**Supplementary file 1b**

|  | **# of spicules** | **Avrg. Length** | **Stdev. Length** | **Avrg. Thickness** | **Stdev. Thickness** | **Avrg. Volume** | **Stdev. Volume** | **Avrg. Area** | **Stdev. Area** |
| --- | --- | --- | --- | --- | --- | --- | --- | --- | --- |
| **Control_48h** | 44 | 497.2 | 62.43 | 3.7 | 0.44 | 6860.28 | 1999.61 | 5822.46 | 1121.57 |
| **Control_72h** | 51 | 697.4 | 71.69 | 4.4 | 0.37 | 14673.2 | 2747.3 | 9896.75 | 1199.63 |
| **Y25_48h** | 93 | 187.93 | 50.18 | 3.92 | 0.53 | 2925.29 | 973.86 | 2324.51 | 606.62 |
| **Y25_72h** | 94 | 276.29 | 81.8 | 4.46 | 0.65 | 5799.48 | 2408.91 | 4002.78 | 1250.25 |

**Supplementary file 1c**

|  | **Y25 48hpf** |
| --- | --- |
| **Control 48hpf** | Length: p=2.2e-63, t=31.0771 |
|  | Thickness: p=0.02, t=-2.342 |
|  | Volume: p=8.1e-32, t=15.5207 |
|  | Area: p=6.3e-50, t=23.6854 |
|  | **Y25 72hpf** |
| **Control 72hpf** | Length: p=3.9e-65, t=30.8792 |
|  | Thickness: p=0.5, t=-0.6798 |
|  | Volume: p=2.2e-43, t=20.1038 |
|  | Area: p=1.2e-58, t=27.4415 |

**Supplementary file 1d**

| **Treatment** | **# replicates** | **# scored embryos** |
| --- | --- | --- |
| **DMSO (control)** | 5 | 176 |
| **LatA >20hpf** | 4 | 152 |
| **LatA >25hpf** | 4 | 140 |
| **Bleb >20hpf** | 4 | 162 |
| **Bleb>25hpf** | 4 | 144 |
| **LatA+Bleb>20hpf** | 3 | 96 |
| **LatA+Bleb>25hpf** | 3 | 114 |
